# A Preliminary Retrospective Study to Assess the Short-Term Safety of Traditional Smooth or Microtextured Silicone Gel-Filled Breast Implants in Korea

**DOI:** 10.3390/medicina57121370

**Published:** 2021-12-16

**Authors:** Sanghyuk Han, Robert Kim, Tae Seob Kim, Jung Heum Park, Seung Soo Kim, Cheol Jeong, Ji Heui Lee

**Affiliations:** 1 Department of Anesthesia and Pain Medicine, Korea Cancer Center Hospital, Seoul 01812, Korea; tkdgur3477@naver.com; 2 Department of Medical and Pharmaceutical Affairs, Doctor CONSULT, Seoul 06296, Korea; mcgcompany@naver.com; 3 The Door Plastic Surgery Clinic, Seoul 06134, Korea; ilovetsv@gmail.com; 4 Mighty Plastic Surgery Clinic, Seoul 06612, Korea; pakjh85@naver.com; 5 Liting Plastic Surgery Clinic, Seoul 06035, Korea; pirate3606@gmail.com; 6 Gangnam JC Plastic Surgery Clinic, Jinju 52703, Gyeongnam, Korea; dcjeong8@naver.com

**Keywords:** breast, breast implantation, Kaplan-Meier estimate, postoperative complications, implant capsular contracture, esthetics

## Abstract

*Background and objectives*: We conducted this preliminary retrospective study to assess the short-term safety of silicone gel-filled breast implants (SGBIs) that are commercially available in Korean women. *Materials and methods* :The current retrospective, observational study was conducted in a total of 2612 patients (*n* = 2612) who underwent augmentation mammaplasty using breast implants at our hospitals between 1 January 2017 and 31 August 2021. *Results*: Overall, there were a total of 248 cases (9.49%) of postoperative complications; these include 112 cases of early seroma, 52 cases of shape deformation, 32 cases of CC, 12 cases of early hematoma, 12 cases of rupture, 12 cases of infection, 12 cases of stretch deformities with skin excess and 4 cases of rippling. Overall complication-free survival of the breast implant was estimated at 1564.32 ± 75.52 days (95% CI 1416.39–1712.32). Then, the Motiva Ergonomix™ SilkSurface showed the longest survival (1528.00 ± 157.92 days [95% CI 1218.48–1837.56]), followed by the BellaGel^®^ SmoothFine (1458.4 ± 65.76 days [95% CI 1329.56–1587.28]), the Sebbin^®^ Sublimity (1322.00 ± 51.20 days [95% CI 1221.64–1422.32]), the BellaGel^®^ Smooth (1138.72 ± 161.28 days [95% CI 822.6–1454.84), the Mentor^®^ MemoryGel™ Xtra (698.4 ± 52.64 days [95% CI 595.28–801.52]) and the Natrelle^®^ INSPIRA™ (380.00 ± 170.88 days [95% CI 45.04–714.96]) in the decreasing order. On subgroup analysis, both the Motiva Ergonomix^TM^ and Mentor^®^ MemoryGel™ Xtra showed no postoperative complications. However, the BellaGel^®^ SmoothFine, Sebbin^®^ Sublimity and BellaGel^®^ Smooth showed incidences of 8.87%, 4.84% and 1.61%, respectively. A subgroup analysis also showed differences in incidences of postoperative complications between microtextured and smooth breast implants (15.18% vs. 16.67%). *Conclusions*: In conclusion, our results indicate that diverse types of an SGBI are commercially available and their safety profile varies according to the manufacturer. Plastic surgeons should consider the safety profile of each device in selecting the optimal types of the device for Korean women who are in need of an implant-based augmentation mammaplasty. However, this warrants a single-surgeon, single-center study with long periods of follow-up.

## 1. Introduction

In the United States, augmentation mammaplasty is currently the most common aesthetic operation. In 2018, a total of 329,914 surgeries were performed; the number of procedures was increased by 15.2% as compared with 2014. Similarly, a total of 1,862,506 surgeries were performed worldwide in 2018; the number of procedures was increased by 27.6% as compared with 2014 [1].

To date, Korean society has undergone great advances in medical technology accompanied by a rapid development of internet-based mass media and social network systems. This has contributed to tremendously increasing the number of patients receiving aesthetic surgeries in Korea. According to the International Society of Aesthetic Plastic Surgery (ISAPS) statistics based on samples of aesthetic plastic surgeons worldwide, Korea is ranked as the first on a per capita basis; 1000 Korean patients receive 13.5 aesthetic surgeries. It can be inferred, however, that the actual number of aesthetic surgeries might be higher as compared with the ISAPS statisticsbecause aesthetic surgeries are not covered by the Korean health insurance system [2].

A recent survey showed that the proportion of people who considered to receive aesthetic surgeries was 14% in 1994, 15% in 2004 and 18% in 2015. Moreover, it also showed that the proportion of patients who actually received aesthetic surgeries was 2% in 1994, 5% in 2004 and 7% in 2015 [2]. Furthermore, it has also been reported that the number of aesthetic breast surgeries accounted for 2.7% of the total number of worldwide cases in 2015. More specifically, there was an increase in the number of patients receiving aesthetic breast surgeries from 58,601 in 2014 to 74,876 in 2015 in Korea [3].

A stringent assessment of the safety of a silicone gel-filled breast implant (SGBI) is mandatory for commercial release of it in the market. Still, however, patients receiving an SGBI are at risks of developing adverse outcomes of an implant-based augmentation mammaplasty; these include reoperation, capsular contracture (CC), breast implant illness, breast implant-associated anaplastic large cell lymphoma (BIA-ALCL), pain, rupture or infection [4,5,6,7,8].

It is expected that the size of global market of an SGBI would be increased to USD 2.2 billion by the year of 2026 after it was estimated at USD 1.43 billion in 2019 [9]. The accurate size of the Korean market of an SGBI remains unknown. However, diverse manufacturers compete with each other in Korea; these include the Polytech Health and Aesthetics (Dieburg, Germany), the Allergan Inc. (Irvine, CA, USA), Groupe Sebbin SAS (Boissy-l’Aillerie, France), the Mentor Worldwide LLC (Santa Barbara, CA, USA), the Sientra Inc. (Rio de Janeiro, Brazil), the Establishment Labs Holdings Inc. (Alajuela, CostaRica), the GC Aesthetics PLC (Apt Cedex, France) and the HansBiomed Co. Ltd. (Seoul, Korea) [10,11]. Of these, the Establishment Labs Holdings Inc. triggered the popularity of a microtextured breast implant when it released the Motiva Ergonomix™ Round SilkSurface in 17 June 2016 [12,13]. Use of a microtextured device has become predominant since that of a macrotextured device was banned by the Korean Ministry of Food and Drug Safety (KMFDS) in 29 August 2019 when it announced the first Korean case of BIA-ALCL to the public [14].

The occurrence of breast implant crisis provided stakeholders in the global industry with an opportunity to become aware of the importance of the safety of a device [15,16]. Likewise, stakeholders in the Korean industry of an SGBI have recently experienced breast implant crises from BIA-ALCL and the first Korean case of a medical device fraud. This led to a ban of textured breast implants and devices from a Korean manufacturer, the HansBiomed Co. Ltd., as mandated by the KMFDS in 29 August 2019 and 13 November 2020, respectively [11,12,13,14]. Therefore, it is allegedly known that only traditional smooth or microtextured breast implants from foreign manufacturers are commercially available currently in Korea; these include the Motiva Ergonomix™ SilkSurface (Establishment Labs Holdings Inc.), the Sebbin^®^ Integrity™, Sublimity™ and Purity™ (Groupe Sebbin SAS), the Eurosilicone Round Collection™ (GC Aesthetics PLC), the Mentor^®^ MemoryGel^TM^ Xtra (Mentor Worldwide LLC.) and the Natrelle^®^ INSPIRA (Allergan Inc.).

Given the above background, we conducted this preliminary retrospective study to assess the short-term safety of traditional smooth or microtextured breast implants that are commercially available in Korean women.

## 2. Materials and Methods

### 2.1. Study Patients and Setting

The current retrospective, observational study was conducted in a cohort of patients who underwent augmentation mammaplasty using breast implants at four hospitals in Korea between 1 January 2017 and 31 August 2021. We included the patients who received primary augmentation mammaplasty using traditional smooth or microtextured breast implants for aesthetic purposes including those receiving a surgery using the BellaGel^®^ breast implants, including the BellaGel^®^ SmoothFine, before 13 November 2020, those who were followed up for ≥1 year and those with available medical records. However, we excluded the patients who received reduction mammaplasty and those lost to follow-up. We therefore evaluated a total of 2612 patients (*n* = 2612) in the current study. We obtained the ethical approval of the current study from the Internal Institutional Review Board (IRB) of the Korea National Institute of Bioethics Policy (IRB approval #: P01-202102-37-056) and conducted it in compliance with the relevant guidelines and applicable laws. However, we failed to receive a written informed consent from the patients because of retrospective nature of the current study.

### 2.2. Treatment Protocol

An implant-based augmentation mammaplasty was performed by four board-certified specialists in plastic and reconstructive surgery, as previously described [10,12].

Surgery was performed under general anesthesia and intravenous sedation via peri-areolar, inframammary fold (IMF) or trans-axillary incisions. Each incision was selected based on desired outcomes, types of breast implants, the degree of augmentation, the anatomical characteristics of patients and patient-surgeon preference. The distance extending from the nipple to the IMF, the size of breast implant and the scope of dissection were determined based on the Ranquist formula. After the dissection, each breast was irrigated using a normal saline 100 cc mixed with H_2_O_2_ solution at a ratio of 1:1, followed by the use of betadine 100 cc. Then, a breast implant was immersed in a normal saline mixed with ceftezole 1 vial and gentamycin 1 ample and then inserted in a subpectoral or subglandular pocket. Placement of a breast implant in a pocket was dependent on its types, the degree of augmentation, characteristics of a patient’s body and our recommendations. Intraoperatively, the patients were intravenously given ceftezole 1.0 g. Incisions were closed using layered sutures in the breast tissue. In addition, the skin was closed using adhesive or surgical tape [10,12].

Postoperatively, the patients were given cefaclor, non-steroidal anti-inflammatory drugs and antacid three times daily for a week. Moreover, they were also recommended to take montelukast sodium 10 mg (Lucast tab.; Wooridul Pharmaceutical Ltd., Seoul, Korea) for a month for the prevention of CC and to wear a compressive garment for three months. Furthermore, they were also recommended to use an upper or lower band, if necessary, and most of them used an upper one for 1–2 months [10,12].

Postoperative course was meticulously monitored during a regular follow-up at 1, 2, 3 and 4 weeks, 3, 6, 9 and 12 months and thereafter [10,12].

### 2.3. Patient Evaluation and Criteria

In our series, we performed a retrospective review of the medical records. Baseline characteristics of the patients include age, sex, height, weight, purposes of surgery (aesthetic augmentation mammaplasty), follow-up period, surface topography (smooth and microtextured surface), volume (≤245, 250–295, 300–345, 350–395 and ≥400 cc) and profile (ultra-high, high, medium and low profiles) of breast implants, the mode of incision (trans-axillary, IMF and peri-areolar incision) and type of pocket (subpectoral and subglandular pocket).

According to a review of literatures, complications of an implant-based augmentation mammaplasty were assessed at a follow-up period of ≥1 year. This is based on previous published studies showing that most cases of CC occurwithin one year of an implant-based augmentation mammaplasty, which is advocated by a systematic review of the 16 published literatures showing that patients were followedup for ≥1 year [17,18]. Time to events (TTEs), such as a patient’s death, recurrence of a cancer or revision to an implant, may serve as an outcomemeasure; the data about such events are analyzed basedon the TTEs of interest [19].

We analyzed the safety of breast implants, for which we evaluated our clinical series of the patients for occurrences of postoperative complications. Moreover, we also performed a subgroup analysis to compare incidences of postoperative complications between the breast implants after considering risk factors of CC. First, it has been reported that use of a trans-axillary incision or a peri-areolar incision is associated with a higher risk of CC as compared with an IMF incision [20,21]. Second, locations of the implant pocket also serve as risk factors of developing CC; a subglandular pocket is commonly associated with higher incidences of CC as compared with a submuscular or dual-plane one [22]. Thus, we excluded the patients receiving a breast implant via a trans-axillary or peri-areolar incision in a subglandular pocket. Finally, we also analyzed survival of a breast implant; it is defined as survivorship of complication-free patients and then calculated as percentage of implants that survive without undergoing revision to or removal of them [10].

### 2.4. Statistical Analysis of the Patient Data

Data was expressed as the number of patients with percentage, mean ± standard deviation or mean ± standard error with 95% confidence intervals (CIs), where appropriate. The cumulative complication-free survival was estimated, and differences in it between the breast implantswere tested for statistical significance using the repeated measures analysis of variance (ANOVA) and Duncan’s post-hoc analysis. This was followed by the log-rank test. Furthermore, the corresponding Kaplan-Meier survival was plotted as a curve. Statistical analysis was carried outusing the SPSS ver. 18.0 for windows (SPSS Inc., Chicago, IL, USA). Statistical significance was set at *p* < 0.05.

## 3. Results

### 3.1. Baseline Characteristics of the Patients

A total of 2612 patients (*n* = 2612) were evaluated in the current study, all of whom were women with a mean age of 31.59 ± 8.32 years old and a mean follow-up period of 14.47 ± 2.46 (range, 12–17) months. Their baseline characteristics are represented in Table 1.

**Figure 1:**
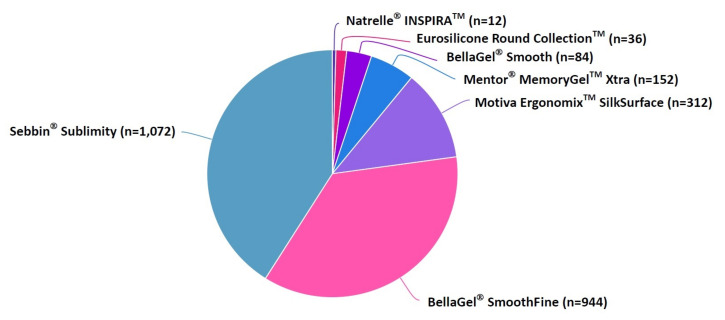
Distribution of breast implants by the manufacturer.

**Figure 2:**
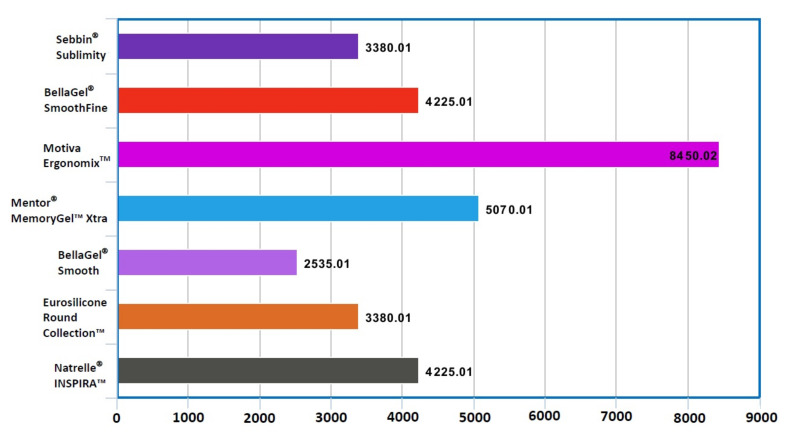
Costs of surgery by the manufacturer of a breast implant.Note: Values are in USD.Costs of surgery varied depending on the manufacturer of a breast implant, ranging from USD 2535.01 to USD 8450.02.

Our clinical series of the patients received augmentation mammaplasty using SGBIs; these include the Sebbin^®^ Sublimity (*n* = 1072) (Groupe Sebbin SAS), the BellaGel^®^ SmoothFine (*n* = 944) (HansBiomed Co. Ltd.), the Motiva Ergonomix™ SilkSurface (*n* = 312) (Establishment Labs Holdings Inc.), the Mentor^®^ MemoryGel™ Xtra (*n* = 152) (Mentor Worldwide LLC), the BellaGel^®^ Smooth (*n* = 84) (HansBiomed Co. Ltd.), the Eurosilicone Round Collection™ (*n* = 36) (GC Aesthetics PLC) and the Natrelle^®^ INSPIRA™ (*n* = 12) (Allergan Inc.) (Figure 1). Costs of surgery varied depending on the manufacturer of a breast implant, ranging from USD 2535.01 to USD 8450.02 (Figure 2). Baseline characteristics of the patients by the breast implants are represented in Table 2.

### 3.2. Incidences of Postoperative Complications by the Breast Implants

Overall, there were a total of 248 cases (9.49%) of postoperative complications; these include 112 cases of early seroma, 52 cases of shape deformation, 32 cases of CC, 12 cases of early hematoma, 12 cases of rupture, 12 cases of infection, 12 cases of stretch deformities with skin excess and 4 cases of rippling (Table 3).

**Figure 3:**
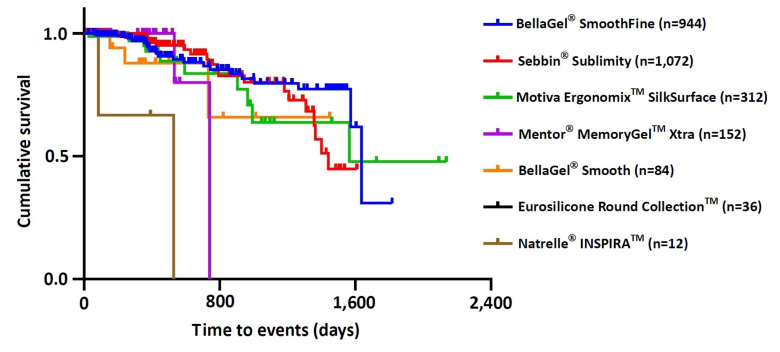
Kaplan-Meier survival curve. The Motiva Ergonomix™ SilkSurface showed the longest survival (1528.00 ± 157.92 days [95% CI 1218.48–1837.56]), followed by the BellaGel^®^ SmoothFine (1458.4 ± 65.76 days [95% CI 1329.56–1587.28]), the Sebbin^®^ Sublimity (1322.00 ± 51.20 days [95% CI 1221.64–1422.32]), the BellaGel^®^ Smooth (1138.72 ± 161.28 days [95% CI 822.6–1454.84), the Mentor^®^ MemoryGel™ Xtra (698.4 ± 52.64 days [95% CI 595.28–801.52]) and the Natrelle^®^ INSPIRA™ (380.00 ± 170.88 days [95% CI 45.04–714.96]) in the decreasing order.

As shown in Table 4, there were significant differences in cumulative incidences of postoperative complications between the breast implants, for which statistical significance was assessed using the log-rank test (χ^2^ = 24.457, *p* = 0.000). Moreover, overall complication-free survival of the breast implant was estimated at 1564.32 ± 75.52 days (95% CI 1416.39–1712.32). Then, the Motiva Ergonomix™ SilkSurface showed the longest survival (1528.00 ± 157.92 days [95% CI 1218.48–1837.56]), followed by the BellaGel^®^ SmoothFine (1458.4 ± 65.76 days [95% CI 1329.56–1587.28]), the Sebbin^®^ Sublimity (1322.00 ± 51.20 days [95% CI 1221.64–1422.32]), the BellaGel^®^ Smooth (1138.72 ± 161.28 days [95% CI 822.6–1454.84), the Mentor^®^ MemoryGel™ Xtra (698.4 ± 52.64 days [95% CI 595.28–801.52]) and the Natrelle^®^ INSPIRA™ (380.00 ± 170.88 days [95% CI 45.04–714.96]) in the decreasing order. The Kaplan-Meier survival curve was plotted in Figure 3.

### 3.3. Results of Subgroup Analysis

**Figure 4:**
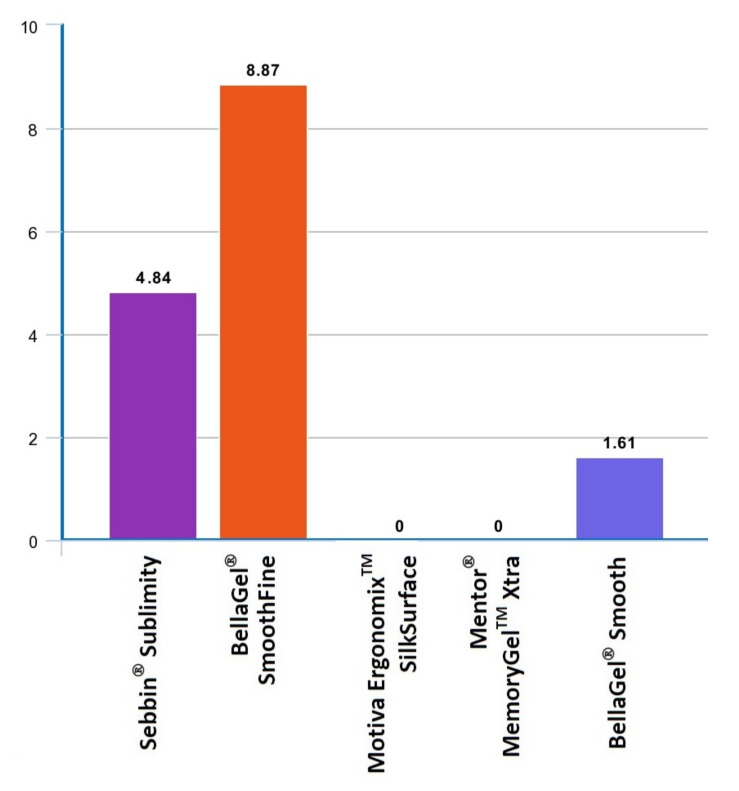
Differences in incidences of postoperative complications between the breast implants on subgroup analysis (*n* = 124). On subgroup analysis, both the Motiva Ergonomix™ SilkSurface and Mentor^®^ MemoryGel™ Xtra showed no postoperative complications. However, the BellaGel^®^ SmoothFine, Sebbin^®^ Sublimity and BellaGel^®^ Smooth showed incidences of 8.87%, 4.84% and 1.61%, respectively.

**Figure 5:**
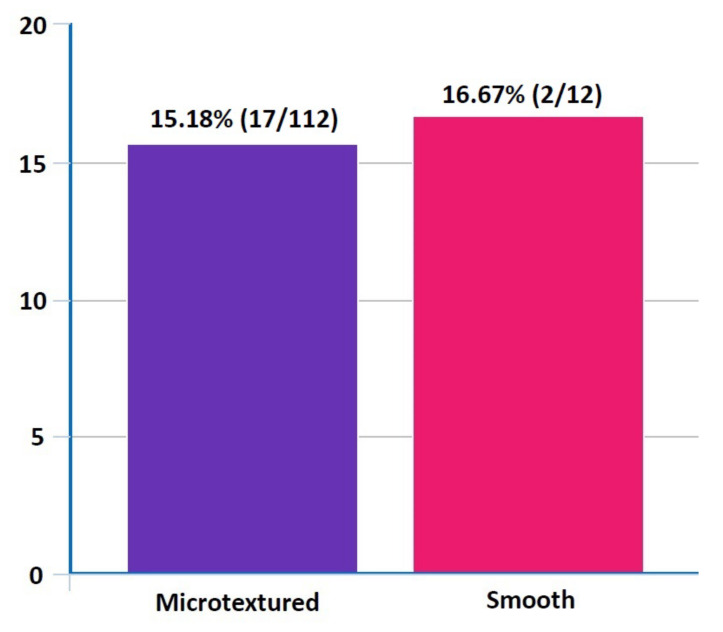
Differences in incidences of postoperative complications between microtextured and smooth breast implants on subgroup analysis (*n* = 124). A subgroup analysis also showed differences in incidences of postoperative complications between microtextured and smooth breast implants (15.18% vs. 16.67%).

On subgroup analysis, both the Motiva Ergonomix^TM^ and Mentor^®^ MemoryGel™ Xtra showed no postoperative complications. However, the BellaGel^®^ SmoothFine, Sebbin^®^ Sublimity and BellaGel^®^ Smooth showed incidences of 8.87%, 4.84% and 1.61%, respectively (Table 5 and Figure 4). A subgroup analysis also showed differences in incidences of postoperative complications between the microtextured and smooth breast implants (15.18% vs. 16.67%) (Figure 5).

## 4. Discussion

Commercially-available SGBIs are equipped with diverse surface topography, such as smooth, microtextured and macrotextured surfaces [23]. Recently, a novel system, the International Organization for Standardization (ISO) 14607:2018, has been developed to classify the surface topography of a device [24,25]. It is based on the surface roughness (Ra) on scanning electron microscopy [25]. Therefore, the surface topography of a device is classified into smooth (Ra < 10 μm), microtextured (10 μm ≤ Ra ≤ 50 μm) or macrotextured (Ra > 50 μm) [26].

Before the voluntary recall of a textured breast implant by the Allergan Inc., the breakdown of the use of a device in the United States was approximately 87% smooth and 13% textured devices. However, there is a considerable difference in the preference for the use of breast implants between the United States and other countries; a textured device is used in approximately 90% of total cases in Europe and Australia [27]. As mentioned earlier, the Korean market of an SGBI is characterized by the popularity of a microtextured device. We found that 90.5% (2364/2612) of the total patients received a microtextured device. This indicates a transition from a textured breast implant to a microtextured device in Korea.

A microtextured device has emerged as an innovative implant technology that may address both CC and BIA-ALCL; a previous macrotextured device has been suggested to actually reduce a risk of CC. However, a causal relationship between a macrotextured breast implant and a risk of BIA-ALCL has popularized the use of a microtextured device [28]. Indeed, contemporary plastic surgeons experience a transition from a macrotextured breast implant to a microtextured device; it is classified as a smooth breast implant according to the latest ISO 14607:2018 definition [28].

Relationship between surface texturing and a decreased risk of CC has been suggested [27,29]. Barnsley GP, et al., performed a meta-analysis of the previous published randomized controlled trials, thus showing that surface texturing had an effect in preventing the occurrence of CC [30]. Moreover, Wong CH, et al. performed a systematic review of published trials, who reported that use of textured implants had a significant correlation with a lower incidence of CC [31]. This has been advocated by another systematic review performed by Schaub TA, et al., who indicated a correlation between textured devices and a lower incidence of CC [18]. Following a comparison of rates of CC between the microtextured and smooth devices, we found that they were higher in microtextured devices as compared with smooth ones (1.35% [32/2364] vs. 0.00% [0/248]). However, the possibility of comparison bias could not be completely ruled out. Further prospective randomized controlled studies are warranted to draw conclusions about differences in rates of CC between the microtextured and smooth breast implants.

An SGBI has undergone evolution; its design is superior to that of its predecessors. To keep up with the state-of-the-art technology, plastic surgeons tend to switch to newer devices [32]. This results in the inconsistency in the distribution of breast implants between them over time. Thus, a heterogeneous mixture of the device is used in diverse surgical settings for varying periods of time [16,33,34]. It is therefore difficult to accurately evaluate the treatment outcomes and safety of an implant-based augmentation mammaplasty. This problem may be compounded by two factors: a sporadic follow-up and a lack of mechanism to ensure a regular follow-up [35]. It would therefore mandatory to accumulate sound clinical data about the safety of an SGBI.

To summarize, our results are as follows: First, overall, there were a total of 248 cases (9.49%) of postoperative complications; these include 112 cases of early seroma, 52 cases of shape deformation, 32 cases of CC, 12 cases of early hematoma, 12 cases of rupture, 12 cases of infection, 12 cases of stretch deformities with skin excess and 4 cases of rippling. Second, overall complication-free survival of the breast implant was estimated at 1564.32 ± 75.52 days (95% CI 1416.39–1712.32). Then, the Motiva Ergonomix™ SilkSurface showed the longest survival (1528.00 ± 157.92 days [95% CI 1218.48–1837.56]), followed by the BellaGel^®^ SmoothFine (1458.4 ± 65.76 days [95% CI 1329.56–1587.28]), the Sebbin^®^ Sublimity (1322.00 ± 51.20 days [95% CI 1221.64–1422.32]), the BellaGel^®^ Smooth (1138.72 ± 161.28 days [95% CI 822.6–1454.84), the Mentor^®^ MemoryGel™ Xtra (698.4 ± 52.64 days [95% CI 595.28–801.52]) and the Natrelle^®^ INSPIRA™ (380.00 ± 170.88 days [95% CI 45.04–714.96]) in the decreasing order. Third, on subgroup analysis, both the Motiva Ergonomix^TM^ and Mentor^®^ MemoryGel™ Xtra showed no postoperative complications. However, the BellaGel^®^ SmoothFine, Sebbin^®^ Sublimity and BellaGel^®^ Smooth showed incidences of 8.87%, 4.84% and 1.61%, respectively. A subgroup analysis also showed differences in incidences of postoperative complications between microtextured and smooth breast implants (15.18% vs. 16.67%).

Limitations of the current study are as follows: First, we conducted the current study under the retrospective design. Second, we evaluated only the patients who had been treated at only four local clinics in Korea. Therefore, the selection bias could not be completely ruled out. Third, we followed up our clinical series of the patients for relatively shorter periods of time. This warrants further long-term follow-up studies. Fourth, we failed to control a bias that may arise from differences in surgical techniques between the surgeons at four different local clinics in Korea. This warrants a single-surgeon, single-center study. Fifth, the patients receiving either the Sebbin^®^ Sublimity or BellaGel^®^ SmoothFine accounted for 77.18% of total cases. Therefore, the possibility of comparison bias could not also be completely ruled out. Sixth, the patients receiving a breast implant via a trans-axillary incision accounted for 92.5% of total cases. Previous studies have shown that use of a trans-axillary incision or a peri-areolar incision is associated with a higher risk of CC as compared with an IMF incision [20,21]. In Asian patients, an IMF incision is not frequently used because it leaves a notable scar; a trans-axillary incision or a peri-areolar incision are frequently used in Asian countries where it is not recommended that a scar be left on the breast [36,37].

## 5. Conclusions

In conclusion, our results indicate that diverse types of an SGBI are commercially available and their safety profile varies according to the manufacturer. Plastic surgeons should consider the safety profile of each device in selecting the optimal types of the device for Korean women who are in need of an implant-based augmentation mammaplasty. However, this warrants a single-surgeon, single-center study with long periods of follow-up.

## Figures and Tables

**Table 1:** Baseline characteristics of the patients (*n* = 2612).

Variables	Values
**Age (years old)**	31.59 ± 8.32
**Sex**	
Men	0 (0.0%)
Women	2612 (100.0%)
**Height (cm)**	162.31 ± 7.66
**Weight (kg)**	51.42 ± 6.03
**FU period** **(months)**	14.47 ± 2.46 (12–17)
**Purpose of surgery**	
Aesthetic augmentation mammaplasty	2612 (100.0%)
**Mode of incision**	
Trans-axillary incision	2416 (92.5%)
IMF incision	124 (4.7%)
Peri-areolar incision	72 (2.8%)
**Type of pocket**	
Subpectoral pocket	1860 (71.21%)
Subglandular pocket	752 (28.79%)
**Volume of breast implant (cc)**	
≤245	52 (2.0%)
250–295	404 (15.5%)
300–345	1280 (49.0%)
350–395	716 (27.4%)
≥400	168 (6.1%)
**Surface topography of breast implant**	
Microtextured	2364 (90.5%)
Smooth	248 (9.5%)
**Profile of breast implant**	
Ultra-high	180 (6.9%)
High	2252 (86.2%)
Medium	168 (6.4%)
Low	12 (0.5%)

Abbreviations: FU, follow-up; IMF, inframammary fold. Values are mean ± standard deviation or the number of cases with percentage, where appropriate.

**Table 2:** Baseline characteristics of the patients by the breast implants.

Variables	Values						
	Sebbin^®^ Sublimity (*n* = 1072)	BellaGel^®^ SmoothFine (*n* = 944)	Motiva Ergonomix™ (*n* = 312)	Mentor^®^ MemoryGel™ Xtra (*n* = 152)	BellaGel^®^ Smooth (*n* = 84)	Eurosilicone Round Collection™ (*n* = 36)	Natrelle^®^ INSPIRA™ (*n* = 12)
**Age (years old)**	31.01 ± 7.26	30.81 ± 8.44	34.19 ± 8.62	28.82 ± 7.83	40.62 ± 11.43	33.00 ± 6.93	45.33 ± 3.21
**Sex**							
Men	0 (0.00%)	0 (0.00%)	0 (0.00%)	0 (0.00%)	0 (0.00%)	0 (0.00%)	0 (0.00%)
Women	1072 (100.00%)	944 (100.00%)	312 (100.00%)	152 (100.00%)	84 (100.00%)	36 (100.00%)	12 (100.00%)
**Height (cm)**	162.64 ± 4.94	161.77 ± 10.81	163.58 ± 5.09	160.74 ± 5.18	163.11 ± 6.00	161.67 ± 4.82	159.67 ± 2.52
**Weight (kg)**	51.15 ± 5.56	51.43 ± 6.01	52.29 ± 5.78	50.37 ± 6.70	55.70 ± 9.02	50.00 ± 6.76	42.00 ± 1.73
**FU period** **(months)**	12.12 ± 0.73	12.19 ± 0.27	12.68 ± 0.58	12.71 ± 0.36	12.67 ± 0.29	12.89 ± 0.04	12.77 ± 0.66
**Purpose of surgery**							
Aesthetic augmentation mammaplasty	1072 (100.00%)	944 (100.00%)	312 (100.00%)	152 (100.00%)	84 (100.00%)	36 (100.00%)	12 (100.00%)
**Mode of incision**							
Trans-axillary incision	1036 (96.64%)	868 (91.95%)	268 (85.90%)	144 (94.74%)	56 (66.67%)	36 (100.00%)	8 (66.67%)
IMF incision	16 (1.49%)	68 (8.62%)	28 (8.97%)	8 (5.26%)	4 (4.76%)	0 (0.00%)	0 (0.00%)
Peri-areolar incision	20 (1.87%)	8 (0.57%)	16 (5.13%)	0 (0.00%)	24 (28.57%)	0 (0.00%)	4 (33.33%)
**Type of pocket**							
Subpectotal pocket	841 (78.45%)	551 (58.37%)	253 (81.09%)	118 (77.63%)	67 (79.76%)	24 (66.67%)	6 (50.00%)
Subglandular pocket	231 (21.55%)	393 (41.63%)	59 (18.91%)	34 (22.37%)	17 (20.24%)	12 (33.33%)	6 (50.00%)
**Volume of breast implant (cc)**							
≤245	4 (1.49%)	2 (0.57%)	3 (3.85)	1 (2.63%)	2 (9.52%)	0 (0.00%)	1 (33.33%)
250–295	36 (13.43%)	28 (12.14%)	16 (20.51%)	11 (28.95%)	5 (23.81%)	4 (44.44%)	1 (33.33%)
300–345	146 (54.48%)	126 (53.39%)	27 (34.62%)	14 (36.84%)	4 (19.05%)	2 (22.22%)	1 (33.33%)
350–395	66 (24.63%)	80 (33.90%)	17 (21.79%)	10 (26.32%)	3 (14.29%)	3 (33.34%)	0 (0.00%)
≥400	16 (5.97%)	0 (0.00%)	15 (19.23%)	2 (5.26%)	8 (38.10%)	0 (0.00%)	0 (0.00%)
**Surface topography of breast implant**							
Microtextured	1072 (100.00%)	944 (100.00%)	312 (100.00%)	0 (0.00%)	0 (0.00%)	36 (100.00%)	0 (0.00%)
Smooth	0 (0.00%)	0 (0.00%)	0 (0.00%)	152 (100.00%)	84 (100.00%)	0 (0.00%)	12 (100.00%)
**Profile of breast implant**							
Ultra-high	0 (0.00%)	0 (0.00%)	180 (57.69%)	0 (0.00%)	0 (0.00%)	0 (0.00%)	0 (0.00%)
High	1040 (97.01%)	848 (90.68%)	96 (30.77%)	152 (100.00%)	72 (85.71%)	36 (100.00%)	0 (0.00%)
Medium	32 (2.99%)	88 (9.32%)	36 (11.54%)	0 (0.00%)	12 (14.29%)	0 (0.00%)	0 (0.00%)
Non-applicable	0 (0.00%)	0 (0.00%)	0 (0.00%)	0 (0.00%)	0 (0.00%)	0 (0.00%)	12 (100.00%)

Abbreviations: FU, follow-up; IMF, inframammary fold. Values are mean ± standard deviation or the number of cases with percentage, where appropriate.

**Table 3:** Distribution of postoperative complications by the breast implants.

	Values						
	Sebbin^®^ Sublimity (*n* = 1072)	BellaGel^®^ SmoothFine (*n* = 944)	MotivaErgonomix^TM^ (*n* = 312)	Mentor^®^ MemoryGel™ Xtra (*n* = 152)	BellaGel^®^ Smooth (*n* = 84)	Eurosilicone Round Collection™ (*n* = 36)	Natrelle^®^ INSPIRA™ (*n* = 12)
Total incidences	88 (8.21%)	92 (9.75%)	40 (12.82%)	8 (5.26%)	12 (14.29%)	0 (0.00%)	8 (66.67%)
Early hematoma	4 (0.37%)	4 (0.42%)	4 (1.28%)	0 (0.00%)	0 (0.00%)	0 (0.00%)	0 (0.00%)
Early seroma	28 (2.61%)	60 (6.36%)	20 (6.41%)	0 (0.00%)	0 (0.00%)	0 (0.00%)	4 (33.33%)
Rupture	4 (0.37%)	4 (0.42%)	0 (0.00%)	0 (0.00%)	0 (0.00%)	0 (0.00%)	4 (33.33%)
Capsular contracture	20 (1.87%)	12 (1.27%)	0 (0.00%)	0 (0.00%)	0 (0.00%)	0 (0.00%)	0 (0.00%)
Rippling	0 (0.00%)	0 (0.00%)	0 (0.00%)	0 (0.00%)	4 (4.76%)	0 (0.00%)	0 (0.00%)
Shape deformation	28 (2.61%)	8 (0.85%)	12 (3.85%)	4 (2.63%)	0 (0.00%)	0 (0.00%)	0 (0.00%)
Infection	4 (0.37%)	4 (0.42%)	0 (0.00%)	0 (0.00%)	4 (4.76%)	0 (0.00%)	0 (0.00%)
Stretch deformities with skin excess	0 (0.00%)	0 (0.00%)	4 (1.28%)	4 (2.63%)	4 (4.76%)	0 (0.00%)	0 (0.00%)

Values are the number of cases with percentage.

**Table 4:** Cumulative incidences of postoperative complications and time-to-events (TTEs) by the breast implants.

Breast Implants	N	*n*	Censored Values	TTE (days)
Total	2612	248	2364 (90.5%)	1564.32 ± 75.52 (1416.39–1712.32)
Sebbin^®^ Sublimity (*n* = 1072)	1072	88	984 (91.8%)	1322.00 ± 51.20 (1221.64–1422.32)
BellaGel^®^ SmoothFine (*n* = 944)	944	92	852 (90.3%)	1458.4 ± 65.76 (1329.56–1587.28)
MotivaErgonomix^TM^ (*n* = 312)	312	40	272 (87.2%)	1528.00 ± 157.92 (1218.48–1837.56)
Mentor^®^MemoryGel™Xtra (*n* = 152)	152	8	144 (94.7%)	698.4 ± 52.64 (595.28–801.52)
BellaGel^®^ Smooth (*n* = 84)	84	12	72 (85.7%)	1138.72 ± 161.28 (822.6–1454.84)
Eurosilicone Round Collection™ (*n* = 36)	36	0	36 (100.0%)	0.00 ± 0.00 (0.00–0.00)
Natrelle^®^ INSPIRA™ (*n* = 12)	12	8	4 (33.3%)	380.00 ± 170.88 (45.04–714.96)

Note: N, total number of cases; *n*, incidence of postoperative complications. Values are the number of cases with percentage or mean ± standard error with 95% confidence intervals, whereappropriate.

**Table 5:** Distribution of postoperative complications by the breast implants on subgroup analysis (*n* = 124).

	Values				
	Sebbin^®^ Sublimity (*n* = 16)	BellaGel^®^ SmoothFine (*n* = 68)	Motiva Ergonomix^TM^ (*n* = 28)	Mentor^®^ MemoryGel™ Xtra (*n* = 8)	BellaGel^®^ Smooth (*n* = 4)
Total incidences	6 (4.84%)	11 (8.87%)	0 (0.00%)	0 (0.00%)	2 (1.61%)
Early hematoma	1 (0.81%)	1 (0.81%)	0 (0.00%)	0 (0.00%)	0 (0.00%)
Early seroma	1 (0.81%)	3 (2.42%)	0 (0.00%)	0 (0.00%)	0 (0.00%)
Rupture	1 (0.81%)	2 (1.61%)	0 (0.00%)	0 (0.00%)	0 (0.00%)
Capsular contracture	0 (0.00%)	0 (0.00%)	0 (0.00%)	0 (0.00%)	0 (0.00%)
Rippling	0 (0.00%)	0 (0.00%)	0 (0.00%)	0 (0.00%)	1 (0.81%)
Shape deformation	2 (1.61%)	3 (2.42%)	0 (0.00%)	0 (0.00%)	0 (0.00%)
Infection	1 (0.81%)	2 (1.61%)	0 (0.00%)	0 (0.00%)	1 (0.81%)
Stretch deformities with skin excess	0 (0.00%)	0 (0.00%)	0 (0.00%)	0 (0.00%)	0 (0.00%)

Values are the number of cases with percentage for the total sample.

## Data Availability

The data presented in this study are available on request from the corresponding author. The data are not publicly available due to privacy reasons.
